# Coregulation of *glutamine synthetase1;2* (*GLN1;2*) and *NADH-dependent glutamate synthase* (*GLT1*) gene expression in Arabidopsis roots in response to ammonium supply

**DOI:** 10.3389/fpls.2023.1127006

**Published:** 2023-02-20

**Authors:** Soichi Kojima, Haruka Minagawa, Chika Yoshida, Eri Inoue, Hideki Takahashi, Keiki Ishiyama

**Affiliations:** ^1^ Graduate School of Agricultural Science, Tohoku University, Sendai, Japan; ^2^ Plant Science Center, RIKEN, Yokohama, Japan; ^3^ Department of Biochemistry and Molecular Biology, Michigan State University, East Lansing, MI, United States

**Keywords:** ammonium response, glutamate synthase (GOGAT), glutamine synthetase (GS), GS/GOGAT, promoter, root, transcriptional (regulation)

## Abstract

Ammonium absorbed by roots is assimilated into amino acids. The glutamine synthetase/glutamate synthase (glutamine 2-oxoglutarate aminotransferase) (GS/GOGAT) cycle is essential to this biological process. In *Arabidopsis thaliana*, *GLN1;2* and *GLT1* are the *GS* and *GOGAT* isoenzymes induced in response to ammonium supply and playing key roles in ammonium utilization. Although recent studies suggest gene regulatory networks involved in transcriptional regulation of ammonium-responsive genes, direct regulatory mechanisms for ammonium-induced expression of *GS/GOGAT* remain unclear. In this study, we revealed that the expression of *GLN1;2* and *GLT1* in Arabidopsis is not directly induced by ammonium but is regulated by glutamine or post-glutamine metabolites produced by ammonium assimilation. Previously, we identified a promoter region required for ammonium-responsive expression of *GLN1;2*. In this study, we further dissected the ammonium-responsive region of the *GLN1;2* promoter and also performed a deletion analysis of the *GLT1* promoter, which led to the identification of a conserved ammonium-responsive region. Yeast one-hybrid screening using the ammonium-responsive region of the *GLN1;2* promoter as a decoy sequence revealed a trihelix family transcription factor DF1 that binds to this region. A putative DF1 binding site was also found in the ammonium-responsive region of the *GLT1* promoter.

## 1 Introduction

Plants absorb nitrogen from the soil to grow (Marschner, 1995). The nitrogen that plants absorb from the soil is either ammonium or nitrate. Nitrate is reduced to ammonium. Ammonium is combined with glutamate and assimilated into glutamine (Marschner, 1995). Ammonium is assimilated primarily in the roots where the glutamine synthetase (GS or GLN) catalyzes this reaction. (Vega-Mas et al., 2019; Kojima et al., 2020). Subsequently, an amino group of glutamine transfers to 2-OG to synthesize glutamate. Glutamate synthase (glutamine 2-oxoglutarate aminotransferase; GOGAT) catalyzes this reaction. Thus, GS and GOGAT are the enzymes for these conjugate reactions. Ammonium assimilation through the GS/GOGAT cycle is the major pathway of nitrogen assimilation in plants (Lea and Miflin, 1974).

Genome sequencing has revealed various isoenzymes of GS/GOGAT in plants. Among them are the isoenzymes expressed in plant roots in response to ammonium supply, such as GS1;2 and NADH-GOGAT1 from rice (Tabuchi et al., 2007) and GLN1;2 and NADH-GOGAT (GLT1) from Arabidopsis (Ishiyama et al., 2004a; Kojima et al., 2014; Konishi et al., 2014). Their transcript and protein accumulation that occurs in response to ammonium manifests the importance of these isoenzymes in ammonium assimilation. Reverse genetic analysis has provided evidence that loss of these ammonium-responsive GS/GOGAT isoenzyme-encoding genes results in reduced ammonium assimilation in plants, particularly in the roots, preventing normal growth (Tamura et al., 2010; Funayama et al., 2013; Konishi et al., 2014; Konishi et al., 2017; Konishi et al., 2018). These results have suggested that ammonium-responsive forms of GS/GOGAT play a central role in the primary assimilation of ammonium in roots (Yamaya and Kusano, 2014). Since the transcripts levels of these isoenzyme-encoding genes increase with ammonium supply, it may be inferred that plants have a transcriptional network to regulate their gene expression in response to ammonium.

Although much of our knowledge has been focused on gene expression networks associated with nitrate as a signal (Konishi and Yanagisawa et al., 2010; Wang et al., 2010; Liseron-Monfils et al., 2013), recent studies also highlight transcriptional networks modulating ammonium responses (Gao et al., 2020; Coleto et al., 2021; Di et al., 2021). WRKY46 is a transcription factor induced by ammonium and regulates ammonium efflux by modulating expression of genes involved in the conjugation of IAA and NUDX9 in Arabidopsis roots (Di et al., 2021). MYB28 and MYB29 are found as transcription factors whose genetic defects increase sensitivity to ammonium (Coleto et al., 2021). WRKY23 is another transcription factor found to be necessary for adaptation of Arabidopsis to high concentrations of ammonium supply (Gao et al., 2020). Notably, despite the necessity of this transcription factor in ameliorating the ammonium toxicity, its loss of function that led to an increased ammonium accumulation in roots had no significant impact on ammonium responsiveness of *GS* gene expression (Gao et al., 2020). Thus, we find genetic evidence to support transcriptional regulation of ammonium response and utilization; however, the information is still fragmental, particularly in regard to mechanisms which directly control *GS/GOGAT* gene expression for ammonium assimilation. Previous studies reveal that rice *NADH-GOGAT1* is expressed in response to ammonium (Hirose et al., 1997; Hirose and Yamaya, 1999). In order to test whether ammonium is a direct signal to induce rice *NADH-GOGAT1* gene expression, they used methionine sulfoximine (MSX) as an inhibitor of GS. When MSX was given simultaneously with ammonium, rice *NADH-GOGAT1* gene expression was not induced. However, when glutamine was given concurrently with MSX, rice *NADH-GOGAT1* gene expression increased. These results suggest that rice *NADH-GOGAT1* expression is not directly induced by ammonium, but by glutamine or its post-glutamine metabolites (Hirose and Yamaya, 1999). In the present study, we examined whether the responsiveness of Arabidopsis *GLN1;2* and *NADH-GOGAT* to ammonium could be regulated by glutamine or post-glutamine metabolites as in rice.

To investigate transcriptional regulatory mechanisms involved in ammonium assimilation, we previously performed a promoter analysis of the *GLN1;2* glutamine synthetase gene of Arabidopsis and identified a 41-bp region required for the ammonium response (Konishi et al., 2017). In the present study, we analyzed the ammonium-responsive promoter regions of *GLN1;2* and *GLT1*, and found a conserved sequence feature for binding a trihelix family transcription factor DF1.

## 2 Materials and methods

### 2.1 Plant growth condition and ammonium treatment

*Arabidopsis thaliana* Columbia-0 (Col-0) accession was used for all experiments. Plants were cultured in a growth chamber controlled at 22°C with 60% relative humidity under 12 hours light and 8 hours dark cycle as described previously (Ishiyama et al., 2004a). The light intensity was 40 µmol m^-2^ s^-1^. Plants grown for two weeks under sterile conditions on MGRL (Molecular Genetics Research Laboratory) medium containing 7 mM nitrate as the nitrogen source (Fujiwara et al., 1992) were subjected to nitrogen starvation for three days prior to the treatment and then transferred to the medium without nitrogen or with 10 mM ammonium chloride (Ishiyama et al., 2004a). Roots of wild-type or transgenic Arabidopsis plants treated with ammonium for 6 hours were used for quantitative real time PCR analysis and visualization of GFP reporter activity. Each medium without nitrogen or with 10 mM ammonium chloride was prepared by adding 10 mM potassium chloride or 10 mM ammonium chloride, respectively, to replace 7 mM potassium nitrate in the MRGL medium.

### 2.2 *GLN1;2*, *GLT1*, and *GLU2* promoter-green fluorescence protein (GFP) fusion gene constructs for transformation of Arabidopsis plants

The fusion gene constructs with various lengths of *GLN1;2* (At1g66200), *GLT1* (At5g53460) and *GLU2* (At2g41220) promoters were generated as follows. The 5’-intergenic regions upstream of coding sequences of *GLN1;2*, *GLT1* and *GLU2* were amplified by polymerase chain reaction (PCR) from genomic DNA of Arabidopsis Col-0 accession and cloned as promoter fragments to generate the GFP reporter fusion constructs. PCR was carried out using KOD plus DNA polymerase (Toyobo, Osaka, Japan) and pairs of forward and reverse oligonucleotide (**Table 1**). The forward primers were designed for amplification of promoter fragments starting 3,583-bp upstream of the translation initiation site of *GLN1;2*, 2,814-bp, 2,200-bp, 2,100-bp, 2,000-bp, 1,930-bp, 1,730-bp, and 1,050-bp upstream of *GLT1*, and 1,358-bp upstream of *GLU2*. Among these forward primers, GLN1;2P3583L_F designed for the amplification of *GLN1;2* promoter region has an overhang of a *Hin*dIII site (AAGCTT) at the 5’-end. The rest of the forward primers designed for the amplification of *GLT1* and *GLU2* promoter regions have an overhang of a *Bam*HI site (GGATCC) at the 5’-end. The reverse primers were designed to have the complementary sequences with the 5’-untranslated regions immediately upstream of the translation initiation sites of *GLN1;2*, *GLT1* and *GLU2*. These reverse primers have an overhang of an *Nco*I site (CCATGG) at the 5’-end. The ATG in the *Nco*I site is the translation initiation site for GFP. The amplified PCR products were subcloned into pCR-Blunt II-TOPO (Thermo Fisher Scientific K.K., Tokyo, Japan), and fully sequenced to confirm the identity. These promoter fragments were then cut out as a *Hin*dIII-*Nco*I fragment (for *GLN1;2*) or *Bam*HI-*Nco*I fragments (for *GLT1* and *GLU2*) and cloned into respective restriction sites of pTH-10KI, replacing the cauliflower mosaic virus (CaMV) 35S promoter, to obtain the promoter:GFP:teminator cassettes. pTH-10KI is the modified version of CaMV35S-synthetic GFP (sGFP, S65T) vector(Chiu et al., 1996; Niwa et al., 1999) and has a full EGFP coding sequence (Takara Bio Inc. Shiga, Tokyo) between the 35S promoter and the nopaline synthase terminator (NosT). Finally, the promoter:GFP : NosT cassettes created in pTH-10KI were cut out as a *Hin*dIII-*Eco*RI fragment (for *GLN1;2*) or *Bam*HI-*Eco*RI fragments (for *GLT1* and *GLU2*) and cloned into pBI101 (Takara Bio Inc.). These binary vector plasmids were introduced into *Agrobacterium tumefaciens* GV3101 (pMP90) by freeze-thaw method as previously described (Ishiyama et al., 2004a). Arabidopsis plants were transformed according to the floral dip method (Clough and Bent, 1998). Transgenic plants were selected on GM medium (Valvekens et al., 1988) containing 50 mg/L kanamycin sulfate. Kanamycin-resistant T2 progenies were used for analyses.

**Table 1:** Primers used for vector construction in this study.

Name	Gene	Direction	Sequence
GLN1;2P3583L_F	GLN1;2	Forward	5’- GAAGCTTTTACCACATTGTTTAATTGTTTCTTAAC-3’
NGP2814L_F	NADH-GOGAT	Forward	5’-GGGATCCTCGATAGATGAGGTGGACAGATTCATAGG-3’
NGP2200L_F	NADH-GOGAT	Forward	5’-GGGATCCTCGTCAACTTTTTGGATGCATAGTTCGAT-3’
NGP2100L_F	NADH-GOGAT	Forward	5’-GGGATCCTAAGAAGTCATTAAAATTATATAATATTA-3’
NGP2000L_F	NADH-GOGAT	Forward	5’-GGGATCCTTAATTCTTGAAAGGGTCAACATTTTGTT-3’
NGP1930L_F	NADH-GOGAT	Forward	5’-GGGATCCTTAAGTATTTAACTAATGTCGTAAGATTA-3’
NGP1730L_F	NADH-GOGAT	Forward	5’-GGGATCCAGCTTGACTATGAAACGTATCAAATTAGT-3’
NGP1050L_F	NADH-GOGAT	Forward	5’-GGGATCCCTTAAATTTCTTAAATTATACATATATAT-3’
NGP_R	NADH-GOGAT	Reverse	5’-GCCATGGTTTTTAGGTTACGGAATCAGCAGTGAGT-3’
FD2P1360_F	Fd-GOGAT2	Forward	5’-CGGATCCGATGGTCTCAAGTTGTCTCTGGCGTTTT-3’
FD2P_R	Fd-GOGAT2	Reverse	5’-GCCATGGGGAATGAAGCTCCTGAGAAGAAACGCCG-3’

### 2.3 Visualizations of GFP in transgenic Arabidopsis roots

GFP visualizations in whole plants transformed with the promoter:GFP gene constructs were performed as described previously (Maruyama-Nakashita et al., 2004). Expression of GFP in transgenic plants was visualized using an image analyzer FluorImager 595 under 488 nm excitation (Molecular Dynamics, Sunnyvale, CA, USA). GFP and autofluorescence of the plant were detected by 530DF30 and 610RG filters, respectively (Maruyama-Nakashita et al., 2005). Relative intensity of GFP signals was quantified with IMAGEQUANT software (Molecular Dynamics). Fluorescence of GFP in transgenic plants were observed under a BX61 microscope equipped with a FV500 confocal laser scanning system and 505-525-nm band pass filter (Olympus, Tokyo, Japan), as described previously (Ishiyama et al., 2004a).

### 2.4 Quantitative real time PCR analysis

Extraction of total RNA, reverse transcription, and real-time PCR were performed as described previously (Maruyama-Nakashita et al., 2004). Total RNA was isolated using RNeasy Plant Mini Kit (Qiagen, Hilden, Germany), and treated with DNaseI (Thermo Fisher Scientific K.K.). Reverse transcription was carried out using Superscript II reverse transcriptase (Thermo Fisher Scientific K.K.) with oligo-d(T)12-18 priming. Real time PCR was carried out using SYBR green PCR master mix and GeneAmp 5700 Sequence Detection System (Applied Biosystems, Foster City, CA, USA). Gene specific primer pairs used are shown in **Table 2**. Calculation of mRNA contents was carried out using *UBQ2* (At2g36170) as a constitutive internal control.

**Table 2:** Primers used for RT-qPCR in this study.

Name	Gene	Direction	Sequence
GFP_RF	GFP	Forward	5’-CTACGGCAAGCTGACCCTGAAGTT-3’
GFP_RR	GFP	Reverse	5’-AGGACCATGTGATCGCGCTTCTC-3’
GLN1;2_RF	GLN1;2	Forward	5’-TGTTAACCTTGACATCTCAGACAACAGT-3’
GLN1;2_RR	GLN1;2	Reverse	5’-ACTTCAGCAATAACATCAGGGTTAGCA-3’
NG_RF	NADH-GOGAT	Forward	5’-AGTTGGGAGAAGGATGAAACCGGGAGG-3’
NG_RR	NADH-GOGAT	Reverse	5’-TTGTAGCTTGGCGTCTTCGTCATCATCC-3’
FG2_RF	Fd-GOGAT2	Forward	5’-GTTGAAGGCACTGGAGATCATTGCTGTG-3’
FG2_RR	Fd-GOGAT2	Reverse	5’-ATCATTGCCCCTTTGCTGCTTCCCGTTT-3’
UBQ2-144F	UBQ2	Forward	5’-CCAAGATCCAGGACAAAGAAGGA-3’
UBQ2-372R	UBQ2	Reverse	5’-TGGAGACGAGCATAACACTTGC-3’

### 2.5 Yeast one-hybrid screening

Yeast one-hybrid (Y1H) screening was performed using the Matchmaker Gold Yeast One-Hybrid Library Screening System (Takara Bio Inc.). The 41 bp ammonium-responsive region (–3,604 to –3,564-bp) found in the *GLN1;2* promoter was used as a bait fragment. The bait fragment was cloned into the pAbAi vector (Takara Bio Inc.). The vector was linearized with *Bbs*I and subsequently transferred to *Saccharomyces cerevisiae* strain Y1H Gold with a LiAc method. Auto-activation was checked by the growth of the bait strain on the medium containing various concentration of Aureobasidin A (AbA). The prey library used in this study was the complete Arabidopsis transcription factor collection (Mitsuda et al., 2010). Approximately 3.7 × 10^4^ transformants were initially screened on SD/−Leu medium containing 500 ng mL^−1^ AbA for 5 days at 30°C to test the possible interaction. Preys were identified from the positive colonies by DNA sequencing.

## 3 Results

### 3.1 Expression profile of three GOGAT genes (*GLU1*, *GLU2* and *GLT1*) in various organs of Arabidopsis

Arabidopsis has three GOGAT genes which are the NADH-dependent GOGAT coded by *GLT1* and two ferredoxin (Fd)-dependent GOGAT coded by *GLU1* and *GLU2* in its genome. The qPCR analysis showed that *GLT1* was the major GOGAT expressed in Arabidopsis roots in addition stems, flowers, and siliques when the plants were grown hydroponically (**Figure S1A**). In contrast, *GLU1* was mainly expressed in young rosette and matured leaves (**Figure S1A**). *GLU2* was expressed in both roots and aboveground tissues, while the amount of the transcripts was lower than the other two GOGAT (**Figure S1A**). Our previous study indicates that *GLN1;2* is the only form of GS showing increased expression in roots in response to ammonium supply among the five cytosolic GS (GS1: *GLN1;1* – *GLN1;5*) and one plastidial GS (GS2: *GLN2*) in Arabidopsis (Ishiyama et al., 2004a). To test whether expression of the three *GOGAT* genes is stimulated by exogenous supply of ammonium, nitrogen-starved Arabidopsis seedlings were exposed to 10 mM ammonium chloride for 6 hours as described previously (Ishiyama et al., 2004a) (**Figure S1B**). Relative to no nitrogen control, the transcript level of *GLT1* encoding the NADH-GOGAT significantly increased in roots in response to ammonium (**Figure S1B**). Thus, the ammonium response of gene expression was similar between *GLT1* (**Figure S1B**) and *GLN1;2* (Ishiyama et al., 2004a). In contrast, the transcript levels of *GLU1* and *GLU2* encoding the Fd-GOGAT did not change when ammonium was supplied to the roots of nitrogen-starved seedlings (**Figure S1B**).

### 3.2 Cell type-specific expression of *GLN1;2* and *GLT1* in Arabidopsis Roots

**Figure 1:**
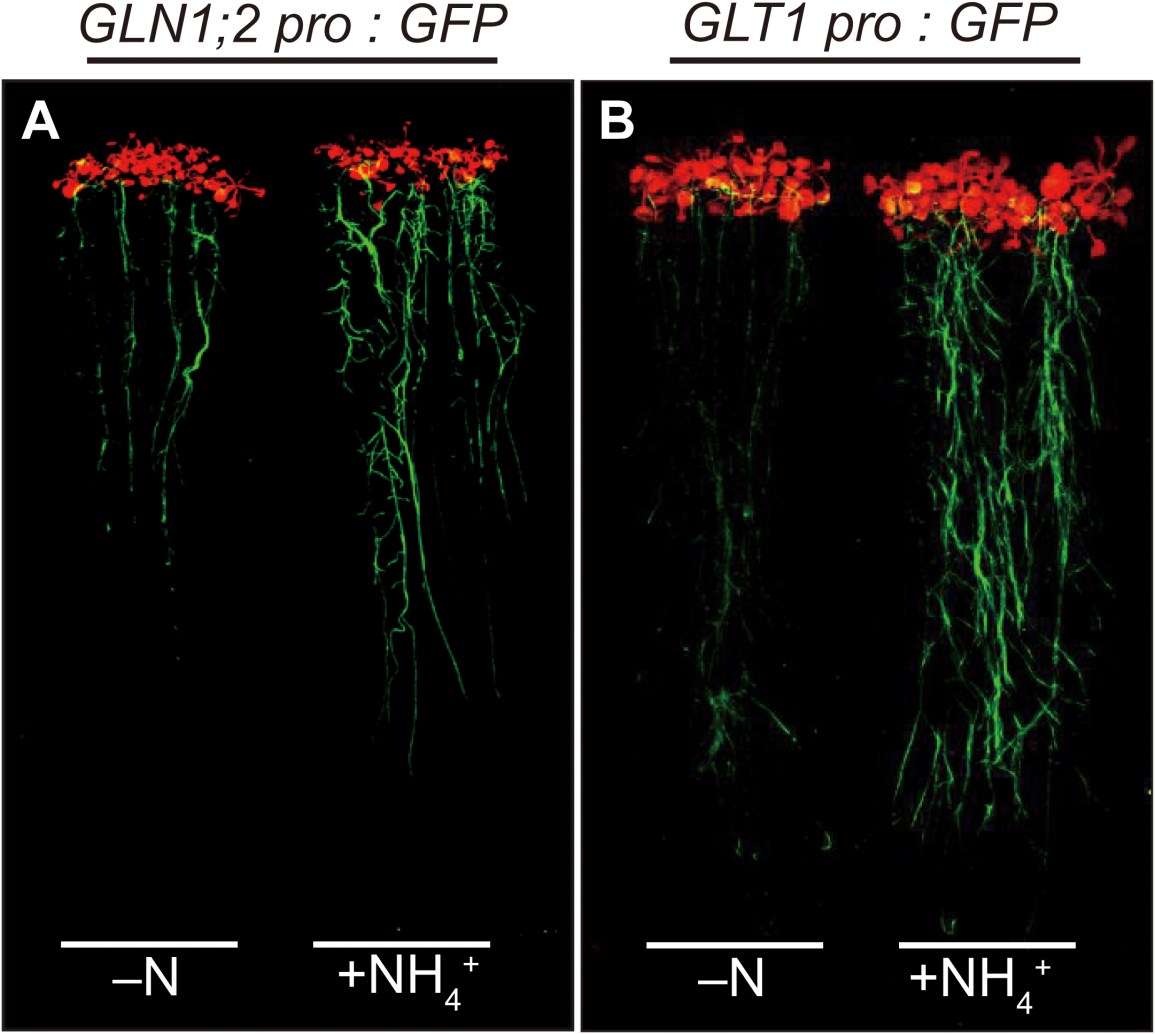
Induction of GFP accumulation in *GLN1;2* promoter:GFP and *GLT1* promoter:GFP lines in response to ammonium supply. The promoter:GFP lines for *GLN1;2* and *GLT1* were germinated and grown on MGRL agar media for 2 weeks, transferred to media without nitrogen (–N) and subjected to nitrogen starvation for 3 days prior to the treatment, and then transferred again to the MGRL media without nitrogen (–N) or with 10 mM ammonium as the sole nitrogen source (+NH_4_^+^). For the promoter constructs, 5,697-bp *GLN1;2* promoter region **(A)** and 2,814-bp *GLT1* promoter region **(B)** upstream of their translation initiation sites were used. The fluorescence of GFP was visualized using FluorImager. At least five independent transgenic lines for each promoter:GFP construct were examined.

**Figure 2:**
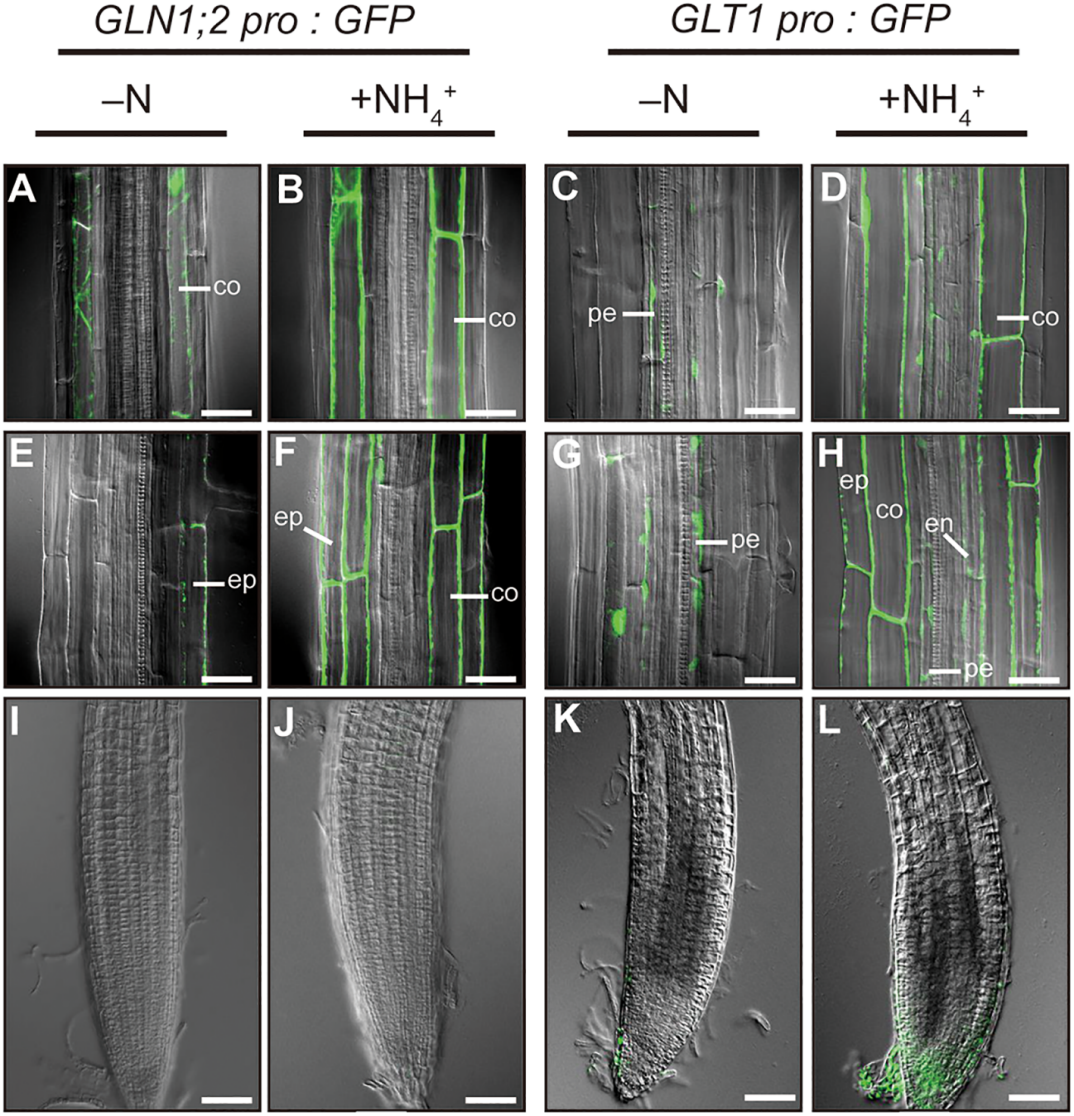
Cell type-specific expression of *GLN1;2* and *GLT1* genes in Arabidopsis roots. The promoter:GFP lines for *GLN1;2* (5,697-bp promoter) and *GLT1* (2,814-bp promoter) were grown and subjected to ammonium treatment as described in **Figure 1**. Whole-mount images from root tips **(I–L)**, elongation zones **(E–H)** and mature zones **(A–D)** of roots were taken by confocal scanning microscopy. At least five independent transgenic lines for each promoter: GFP construct were examined. co, cortex; en, endodermis; ep, epidermal; pe, pericycle cell. Bar = 50 µm **(I–L)** and 25 µm **(A–H)**.

To investigate cell-type specific ammonium responses of *GLN1;2* and *GLT1* expression in Arabidopsis, transgenic lines carrying the *GLN1;2* or *GLT1* promoter:GFP fusion gene constructs were grown with or without ammonium supply (**Figure 1**). Five independent transgenic lines expressing GFP under control of 5,697-bp *GLN1;2* promoter (Konishi et al., 2017) and 2,814-bp *GLT1* promoter, respectively, were analyzed to assess their ammonium response in roots. Fluorescence scanning of whole seedlings revealed marked increase in the intensity of GFP signals in roots of both transgenic lines with 6 hours of ammonium treatment following nitrogen starvation (**Figure 1**). Based on these findings, we carried out confocal laser microscopy analyses of these transgenic lines to identify cell types of the roots where the promoter activity of *GLN1;2* and *GLT1* are present and can be seen as fluorescence of GFP. Under the control of the *GLN1;2* promoter, only faint signals of GFP were detected in the epidermis of elongation zone as well as in the cortex of mature zone of roots when there was no nitrogen supply (**Figures 2A, E**). In contrast, strong GFP signals were detected upon ammonium supply, located specifically in the cortex of the mature zone (**Figure 2B**) and in the epidermis and cortex of the elongation zone of roots (**Figure 2F**). In the *GLT1* promoter:GFP lines, fluorescent signals derived from GFP expression were present in the pericycle cells of both elongation and mature zones of roots with no nitrogen supply (**Figures 2C, G**), although even stronger signals were detected in the cortex of matured zone (**Figure 2D**) and in the epidermis and cortex of elongation zone of roots (**Figure 2H**) upon ammonium supply. In root tips, almost no or very faint GFP signals were detected in both promoter:GFP lines (**Figures 2I–L**). These results show that both *GLN1;2* and *GLT1* are expressed in the surface cell layers of roots, particularly in the epidermis and cortex of roots in response to ammonium supply.

### 3.3 MSX treatment

Since glutamine (or a post-glutamine metabolite) has been shown to induce *NADH-GOGAT1* expression in rice (Hirose and Yamaya, 1999), we used methionine sulfoximine (MSX), an inhibitor of GS, to investigate whether Arabidopsis *GS/GOGAT* also fluctuates in expression depending on glutamine or a post-glutamine metabolite. The qPCR analysis was carried out in the presence or absence of 10 mM MSX. As shown previously (Ishiyama et al., 2004a) and in **Figure S1**, a substantial increase in *GLN1;2* and *GLT1* expression was observed at 6-h after ammonium supply in the roots, but this increase was not observed when MSX was added prior to ammonium supply (**Figure S2**). Over approximately 3-fold increase in *GLN1;2* and *GLT1* expression was observed after glutamine supply regardless of the MSX pre-treatment (**Figure S2**). The expression of *GLU2* and *UBQ2* was stable after ammonium and glutamine supply with or without MSX (**Figure S2**).

### 3.4 Identification of ammonium-responsive regions (ARR) in *GLT1* promoter

**Figure 3:**
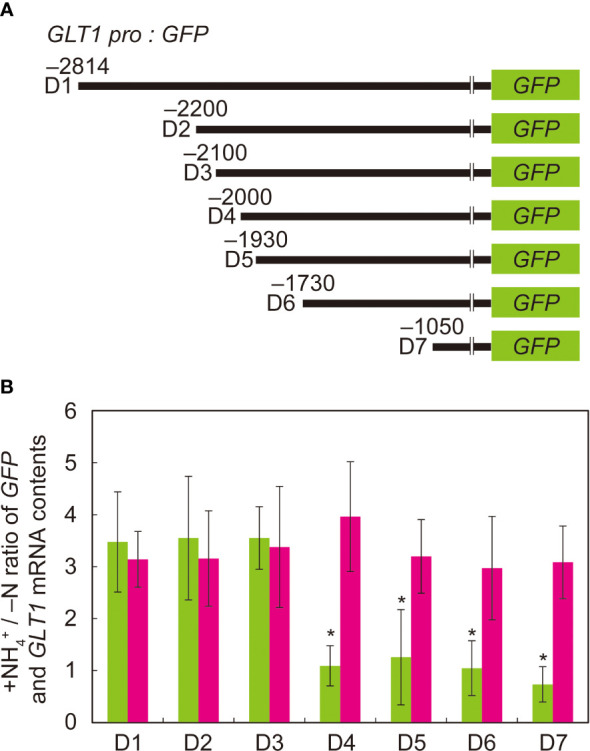
Deletion analysis of ammonium-responsive region of *GLT1* promoter. **(A)** Schematic chart of the 5’ deletion of the *GLT1* promoter fused to GFP. **(B)** RT-qPCR analysis of *GLT1* and *GFP* mRNA levels in root tissues of *GLT1* promoter: GFP lines. Plants were grown and subjected to ammonium treatment as described in **Figure 1**. The letters denote the names of promoter:GFP fusion constructs with various length of *GLT1* promoters described in **(A)**: (D1) 2,814-bp, (D2) 2,200-bp, (D3) 2,100-bp, (D4) 2,000-bp, (D5) 1,930-bp, (D6) 1,730-bp, and (D7) 1,050-bp. The ammonium-responsive accumulations of *GLT1* (magenta column) and *GFP* (green column) transcripts were determined based on their relative abundance between the ammonium-treated and no nitrogen control samples (+NH_4_^+^/-N). Means of five to ten independent RNA samples and standard deviations are indicated in the bar graph. Significant differences between *GLT1* and *GFP* were identified by Student’s t-test are indicated with asterisks.

To determine the “ammonium-responsive region” (ARR) in the 5’-intergenic region upstream of the translation initiation site of *GLT1*, we created a 5’-deletion series of *GLT1* promoter:GFP fusion constructs and introduced them into Arabidopsis by Agrobacterium infection. D1, D2, D3, D4, D5, D6 and D7 are the fusion constructs generated to have the *GLT1* promoter regions from positions –2,814, –2,200, –2,100, –2,000, –1,930, –1,730 and –1,050 bp, respectively, cloned in front of GFP (**Figure 3A**). Transgenic plants generated with these constructs were grown with or without ammonium supply. Among them, D1, D2 and D3 showed 3.5-fold increase in GFP mRNA levels on ammonium compared to no nitrogen control (**Figure 3B**). In contrast, D4, D5, D6 and D7 showed no induction of GFP expression in response to ammonium (**Figure 3B**). The endogenous *GLT1* mRNA levels increased in response to ammonium treatment by 3- to 4-fold relative to no nitrogen control in all *GLT1* promoter:GFP lines (**Figure 3B**). Thus, the ammonium-responsive induction of endogenous *GLT1* transcript expression was consistent with our previous observation (Konishi et al., 2014).

### 3.5 Trihelix family transcription factor DF1 binds ARR

**Figure 4:**
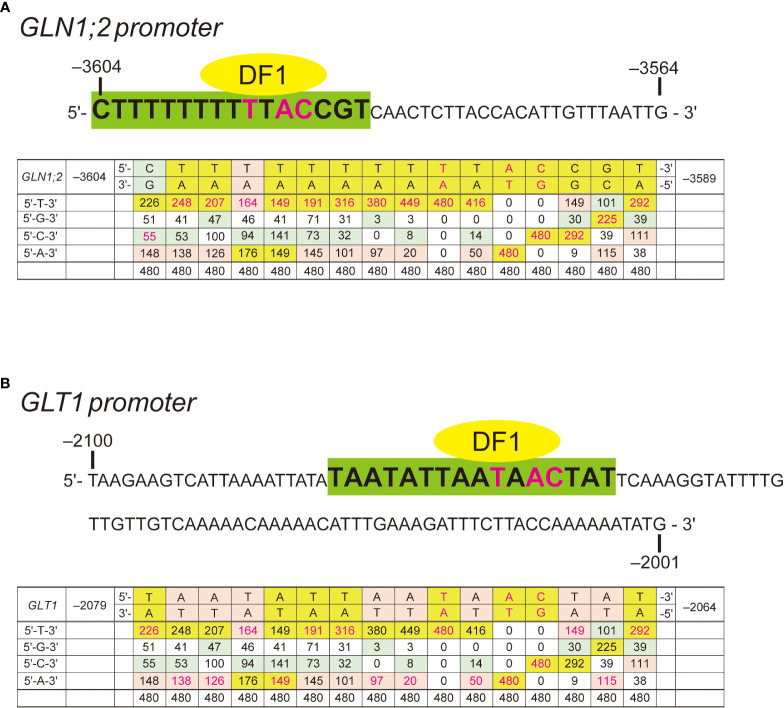
Ammonium responsive regions (ARRs) with DF1 binding sequence signatures. **(A)** The 41-bp (–3,604 to –3,564) ARR in the *GLN1;2* promoter. A predicted DF1-binding motif (–3604 CTTTTTTTTTTACCGT –3,589) is highlighted in green. **(B)** The 100-bp (–2,100 to –2,001) ARR in the *GLT1* promoter. A predicted DF1-binding motif (–2,079 TAATATTAATAACTAT –2,064) is highlighted in green. The frequency matrix of DF1 was compared to sequences predicted to be recognized by DF1 found within the ammonium-responsive promoter regions of *GLN1;2* **(A)** and *GLT1* **(B)**. Frequency matrix was obtained from JASPAR (Castro-Mondragon et al., 2021).

In our previous study, we found the ARR in the *GLN1;2* promoter between the positions –3,604 and –3,564 bp upstream of the translation initiation site (Konishi et al., 2017). To explore potential transcription factor binding sites in the ARR, we performed yeast one-hybrid screening. A GAL4-AD library for yeast one-hybrid system consisting only of Arabidopsis transcription factors (Mitsuda et al., 2010) was screened using the 41-bp region of the *GLN1;2* promoter required for the ammonium response (*i.e.*, –3,604 to –3,564 bp) as a decoy sequence. Approximately 3.7 × 10^4^ yeast colonies were screened, and 24 positive clones were obtained, of which 22 gave the insert sequence information (**Table 3**). Of these 22 Arabidopsis genes identified, 14 were of the AHL family and 3 were DF1. Other transcription factors such as TT16, TTG1, ZFP3, ZFHD2, and LBD22 were also identified as positive clones. Among these transcription factors, a DF1-binding sequences was found between the positions –3,604 and –3,564 bp of *GLN1;2* promoter (**Figure 4A**). The DF1-binding sequence was also found within the ARR of *GLT1* promoter (**Figure 4B**). AHL and ZFHD2 were predicted to recognize and bind TTTAATT in the decoy sequence (**Figure 4A**). In contrast, binding sites for TT16, TTG1, ZFP3, ZFHD2 and LBD22 were not found in the decoy sequence (**Figure 4A**).

**Table 3:** Genes found in yeast one-hybrid screening with ammonium responsive region found in *GLN1;2* promoter.

Clone ID of yeast colonies	Locus ID	name
1	AT5G23260	TT16
2	AT4G00200	AHL7
3	AT1G14490	AHL28
4	AT1G76880	DF1
5	AT1G14490	AHL28
12	AT4G35390	AHL25
13	AT3G55560	AHL15
19	AT1G76880	DF1
11	AT3G55560	AHL15
15	AT4G17800	AHL23
17	AT5G24520	TTG1
18	AT1G14490	AHL28
20	AT1G20900	AHL27
21	unknown	IV
22	AT5G25160	ZFP3
23	AT1G14490	AHL28
24	AT4G17800	AHL23
26	ST1G76880	DF1
27	AT3G55580	AHL15
28	AT3G04570	AHL19
29	AT1G94490	AHL28
31	AT5G65410	ZFHD2
32	AT3G13850	LBD22
33	unknown	IV

**Figure 5:**
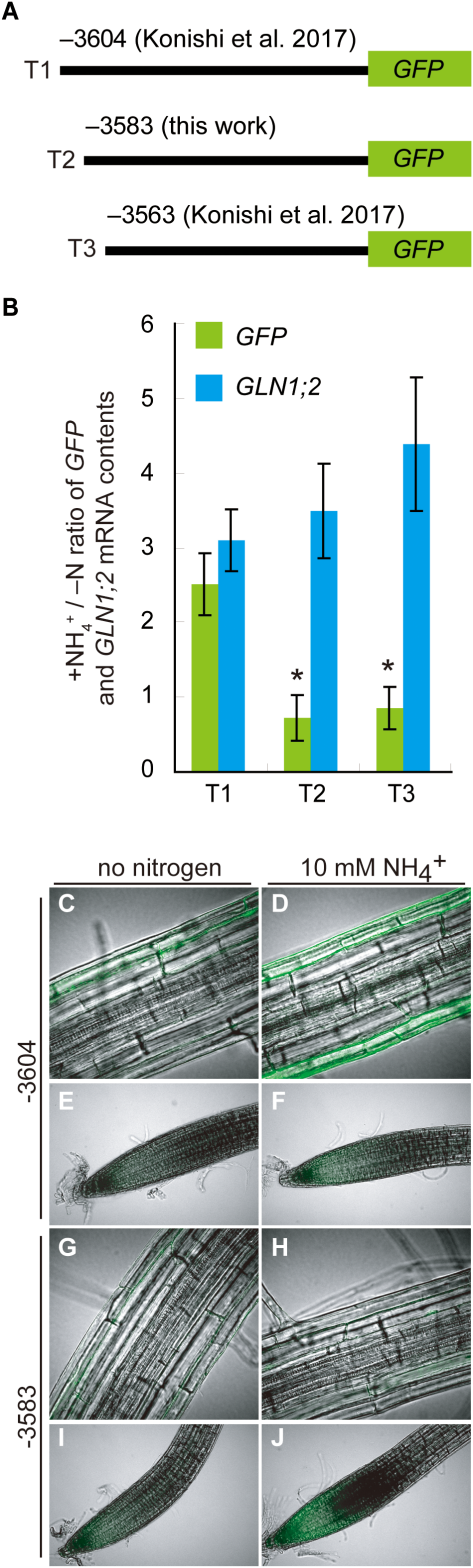
Deletion analysis of ammonium responsive region of *GLN1;2* promoter. **(A)** Schematic chart of the 5’ deletion of the *GLN1;2* promoter fused to GFP. **(B)** RT-qPCR analysis of *GLN1;2* and *GFP* mRNA levels in root tissues of *GLN1;2* promoter:GFP lines. Plants were grown and subjected to ammonium treatment as described in Figure 1. The letters denote the names of promoter:GFP fusion constructs with various length of *GLN1;2* promoters described in **(A)**: (T1) 3,604-bp, (T2) 3,583-bp, and (T3) 3,563-bp. The ammonium-responsive accumulations of *GLN1;2* (cyan column) and *GFP* (green column) transcripts were determined based on their relative abundance between the ammonium-treated and no nitrogen control samples (+NH_4_^+^/–N). Means of five to ten independent RNA samples and standard deviations are indicated. Significant differences between *GLN1;2* and *GFP* were identified by Student’s t-test are indicated with asterisk symbols. **(C–J)** Cell type-specific expression of GFP in promoter:GFP lines with 3,604-bp and 3,583-bp *GLN1;2* promoter regions. Plants were subjected to ammonium treatment **(D, F, H, J)** or no nitrogen media **(C, E, G**, **I)** as described in **Figure 1**. Whole-mount images from root tips **(E, F, I**, **J)** and elongation zones of roots **(C, D, G**, **H)** were taken by confocal scanning microscopy.

To gain further insights into this ARR, we generated promoter:GFP lines designated T2 with a construct which has the 5’-promoter region of *GLN1;2* starting from the position –3,583 bp and lacking the DF1-binding site (**Figures 4A**, **5A**). T1 and T3 lines generated previously with the *GLN1;2* promoter region with or without the ARR starting from –3,604 and –3,564 bp, respectively, were used in parallel to study the ammonium responsiveness of GFP reporter expression (Konishi et al., 2017). Among these *GLN1;2* promoter:GFP lines, only the T1 lines showed a significant increase in GFP mRNA accumulation on ammonium relative to no nitrogen control (**Figure 5B**). In contrast, in the T2 and T3 lines, the ammonium treatment rather resulted in decreasing GFP mRNA accumulation levels down to 0.6- to 0.8-fold of no nitrogen control (**Figure 5B**). In all these *GLN1;2* promoter:GFP lines, the endogenous *GLN1;2* mRNA levels increased upon ammonium treatment by 3.0 to 4.4-fold relative to no nitrogen control (**Figure 5B**). At the cell-type levels, T1 responded to ammonium and expressed GFP in the epidermis of roots (**Figure 5D**), while T2 did not (**Figure 5H**). In both T1 and T2, the root tip did not respond strongly to ammonium (**Figures 5E, F, I, J**). The cell-type specific patterns of GFP expression identified in T1 lines resembled those with the –5,697 bp *GLN1;2* promoter (**Figure 2**), as both demonstrated strong ammonium response in the epidermis of elongation zone of roots, while they were not exclusively identical in all cell types. These results indicated that the ARR for ammonium-inducible expression is located between the positions –3,604 and –3,583 bp in the *GLN1;2* promoter and between –2,100 and –2,001 bp in the *GLT1* promoter, respectively.

## 4 Discussion

Plants show different adaptations to ammonium environments even within a species, and their responses to ammonium environments are genetically diverse (Sarasketa et al., 2014; Zhou et al., 2021). Arabidopsis thrives in oxidative soils, where nitrate is the primary source of nitrogen (Miller et al., 2007). However, some ecotypes of Arabidopsis native to various geographical locations are adapted to ammonium environments (Sarasketa et al., 2014; Yasuda et al., 2017). Previously, we compared the Arabidopsis ecotypes and found three important mechanisms for adaptation to ammonium environments: 1) the rapid response of GS/GOGAT in roots when ammonium is supplied (Yasuda et al., 2017); 2) the development of lateral roots by suppressing main root elongation when ammonium is supplied (Kojima, 2018; Sasaki and Kojima, 2018); and 3) the maintenance of a high capacity for low-affinity ammonium transport (Yasuda et al., 2017). In this study, we focused on ammonium-responsive transcriptional regulation of GS/GOGAT, particularly of *GLT1* encoding the NADH-dependent GOGAT and *GLN1;2* encoding the cytosolic GS, the two key enzymes for ammonium assimilation in Arabidopsis roots (**Figures 1**, **2**).

The tissue-specific expression patterns of Arabidopsis genes encoding GOGAT isoenzymes indicate that *GLT1* is the most highly expressed GOGAT in Arabidopsis roots (**Figure S1**). Of two Fd-dependent GOGAT, *GLU2* is lowly expressed but *GLU1* transcript was hardly detected in roots (**Figure S1**). *GLT1* is the ammonium-inducible form of GOGAT (**Figures 1–3**, **S1B**); however, this transcript accumulation is abolished in the presence of a GS inhibitor methionine sulfoximine (MSX) but restored by supplying glutamine (**Figue S2B**). These results suggest that, as with *GLN1;2*, *GLT1* expression is not directly induced by ammonium, but is rather dependent on glutamine or post-glutamine metabolites (**Figure S2**), as well as *OsGS1;2*, *OsNADH-GOGAT1*, and *OsAS1* genes in rice (Hirose et al., 1997; Yabuki et al., 2017; Ohashi et al., 2018). In contrast to *GLT1*, *GLU2* does not respond to ammonium in roots (**Figures S1**–**S3**). Among these GOGAT, the NADH-GOGAT encoded by *GLT1* is suggested as the isoenzyme playing a central role in supplying substrates to GS when ammonium is supplied as the nitrogen source (Konishi et al., 2014). The results shown in our present study support this idea, as the ammonium-supplied Arabidopsis roots display an increased promoter activity of *GLN1;2* and *GLT1* in the epidermis and cortex in response to ammonium supply, and these cell types where the promoters of the two genes were active overlap significantly (**Figures 1**, **2**). GS/GOGAT expression in the root surface cell layers in response to ammonium may prevent ammonium from being transported to the vascular tissues (Ishiyama et al., 1998; Konishi et al., 2017). The increase in mRNA accumulation can be attributed to increased transcriptional activity and increased mRNA stability (Hirose and Yamaya, 1999). We found in this study that the increase in *GLT1* mRNA levels on ammonium supply is promoter-dependent and likely due to increased transcriptional activity (**Figures 1**–**3**). NADH-GOGAT1 in rice is also transcriptionally activated upon ammonium supply (Hirose and Yamaya, 1999), and as a result, mRNA (Ishiyama et al., 2003) and protein (Ishiyama et al., 1998) accumulate in root surface cell populations. The expression pattern and ammonium response of NADH-GOGAT appear physiologically relevant and conserved across the plant species.

We have previously shown that a 41-bp sequence in the *GLN1;2* promoter is important for this GS isoenzyme to respond to ammonium (Konishi et al., 2017). In this study, we show that the first 20 bp of the 41 bp ARR of the *GLN1;2* promoter is particularly important. A *GLN1;2* promoter lacking this region was unable to express GFP in the root surface cell layers in response to ammonium (**Figure 5**). The mode of accumulation of ammonium-responsive GS (OsGS1;2) isoenzymes in root surface cells in response to ammonium is likely a common feature shared in rice (Ishiyama et al., 2004b) and Arabidopsis (**Figures 2**, **5**). In this study, we isolated several transcription factors by yeast one-hybrid screening using the ARR of *GLN1;2* as a decoy sequence. Our findings implicate that ARRs of GS/GOGAT involved in assimilation of ammonium in roots contain a conserved sequence signature to which DF1 binds (**Figure 4**). DE1 BINDING FACTOR1 (DF1) is a transcription factor that has been shown to regulate adhesive polysaccharides in seeds (Xu et al., 2022) and contribute to root hair formation in roots (Shibata et al., 2018; Shibata et al., 2022). The mucilage surrounding hydrated Arabidopsis seeds is a extracellular matrix composed mainly of the pectic polysaccharide rhamnogalacturonan I (RG-I). DF1 physically interacts with GLABRA2 (GL2) and both proteins transcriptionally regulate the expression of the RG-I biosynthesis genes MUCILAGE MODIFIED4 (MUM4) and GALACTURONOSYLTRANSFERASE-LIKE5 (GATL5) (Xu et al., 2022). The expression of DF1 and GL2 is directly regulated by TRANSPARENT TESTA GLABRA2 (TTG2) and, in turn, DF1 directly represses the expression of TTG2 (Xu et al., 2022). Shibata et al. (2018) reported that a basic helix-loop helix transcription factor ROOT HAIR DEFECTIVE 6-LIKE 4 (RSL4) promotes, but DF1 and a trihelix transcription factor GT-2-LIKE1 (GTL1) repress root hair growth in Arabidopsis. In addition, transcriptional analysis combined with genome-wide chromatin-binding data showed that DF1 and GTL1 directly bind the RSL4 promoter and regulate its expression to repress root hair growth (Shibata et al., 2018). DF1 is also a transcription factor involved in regulation of nitrogen metabolism (Gaudinier et al., 2018). The networks of transcriptional responses involved in nitrogen utilization in Arabidopsis elucidated by Gaudinier et al. (2018) indicate holistic interactions between transcription factors and promoters of genes involved in nitrogen transport, metabolism, signaling, amino acid metabolism, carbon metabolism, carbon transport, tissue growth, and hormone responses. Their data suggest that DF1 binds to the promoter regions of genes related to nitrogen metabolism, such as NR, NiR, AS1, and nitrate transporter; a group of genes known to respond to ammonium (Kan et al., 2015; Yang et al., 2015; Subudhi et al., 2020; Liang et al., 2021). We predict that DF1 would be a candidate of transcription factors involved in the regulation of *GLN1;2* and *GLT1*, which has not been reported previously (Gaudinier et al., 2018). The promoter regions for *GLN1;2* and *GLT1* used by Gaudinier et al. (2018) are roughly 2 kbp, not including the ARR found in our present study.

In our yeast one-hybrid screening, various homologs of the AHL family transcription factor were repeatedly isolated. However, we consider it unlikely that AHLs are directly involved in modulating the ammonium responsiveness of *GLN1;2*. This is because AHLs are predicted to bind TTTAATT, which is located within a non-essential region downstream of the ARR of *GLN1;2*. ZFHD2 is also predicted to bind TTTAATT. ZFHD2, like AHL, is not expected to be a transcription factor directly involved in ammonium response. TTG1 and LBD22 are known to bind bHLH. The ammonium-responsive sequence of *GLN1;2* used a decoy in our yeast one-hybrid screening contains a bHLH binding sequence CAACTC (Konishi et al., 2017). It is possible that TTG1 and LBD22 interacted with the decoy using yeast bHLH as a scaffold. No DNA motifs for TT16 and ZFP3 binding were found within the ARR of *GLN1;2*. Future analysis of mutants and overexpressors of these transcription factors will help better understand how they interact with *GLN1;2* and *GLT1* promoter regions to modulate expression of these key enzymes involved in ammonium utilization.

## Data availability statement

The raw data supporting the conclusions of this article will be made available by the authors, without undue reservation.

## Author contributions

KI, HT, and SK contributed to conception and design of the study. KI and EI performed the qPCR analysis. KI, EI, and SK performed microscopic analysis. KI, EI, and HT prepared for whole mount GFP image. CY, HM, and SK performed yeast one hybrid screening. KI and SK performed promoter analysis. KI and SK wrote the first draft of the manuscript. HT edited the manuscript. All authors contributed to the article and approved the submitted version.
