# Supplementary material for: Coregulation of glutamine synthetase1;2 (GLN1;2) and NADH-dependent glutamate synthase (GLT1) gene expression in Arabidopsis roots in response to ammonium supply
Source: Front Plant Sci. 2023 Feb 20;14:1127006. doi: 10.3389/fpls.2023.1127006 (PMC9986259; doi:10.3389/fpls.2023.1127006)
Supplement: Supplementary file 1 [file Presentation_1.pdf]

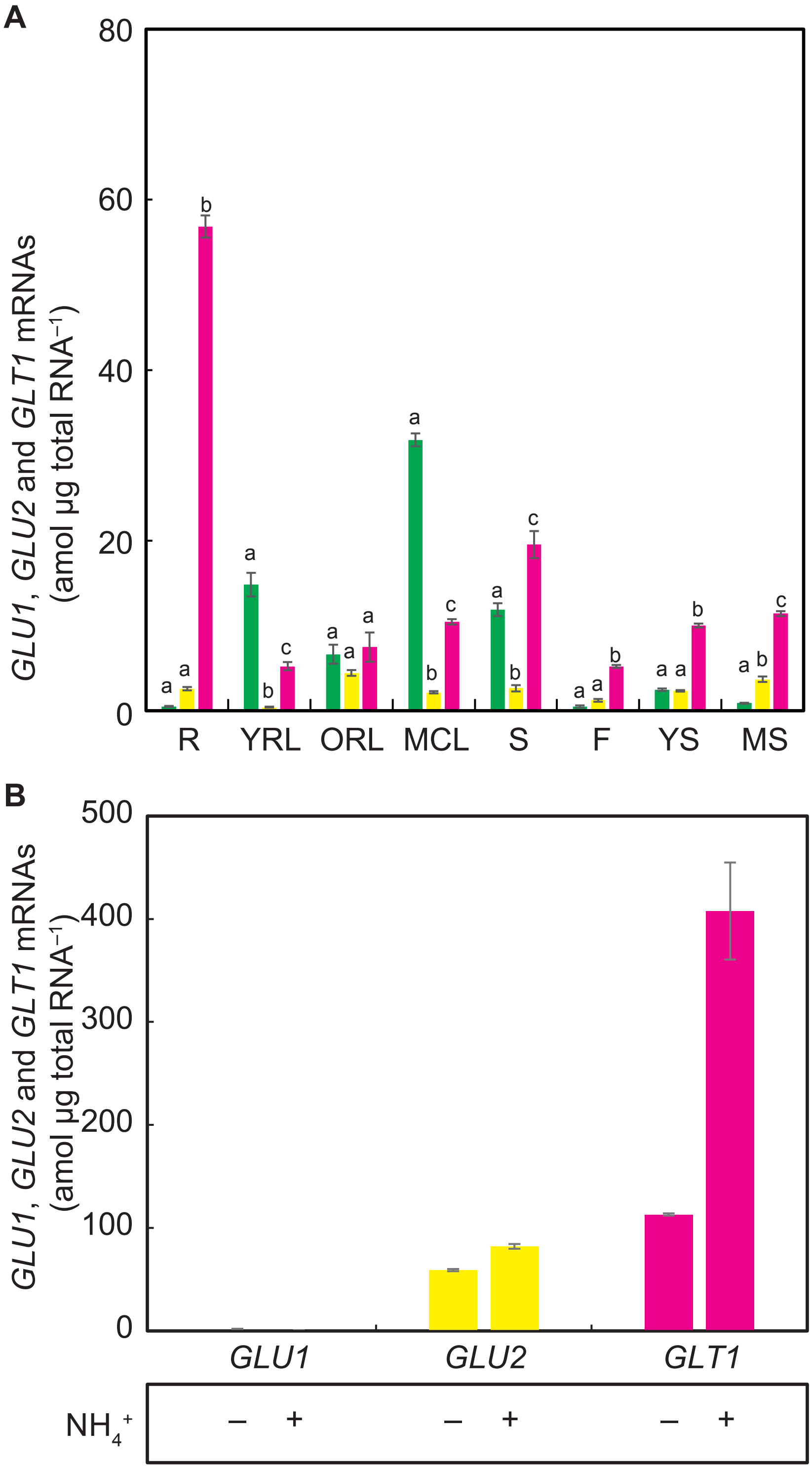


**Figure S1. Comparison of glutamate synthase gene expression levels**

(a) RT-qPCR analysis of gene expression levels of *NADH-GOGAT* (*GLT1*, magenta), *Fd-GOGAT1* (*GLU1*, green), and *Fd-GOGAT2* (*GLU2*, yellow) in different organs. R, root; YRL young rosette leaves; ORL old rosette leaves; MCL mature carline leaves; S stem; F flower; YS young silique; MS mature silique. Arabidopsis were seeded on rockwool and grown on MGRL medium (Naito et al. 1994). Means of three independent RNA samples and standard deviations are indicated. One-way ANOVA analysis was performed and followed by Bonferroni as post-hoc. Letters denote statistically significant differences between the treatment groups and no nitrogen control.

(b) Comparison of ammonium responsiveness of Arabidopsis GOGAT in roots. Two-week-old Arabidopsis plants (accession Col-0) grown on MGRL agar media were subjected to nitrogen starvation for 3 days prior to the treatment. Pre-cultured plants were treated for 6 hours on modified MGRL media without nitrogen (–) or with 10 mM ammonium as the sole nitrogen source ($NH_4^+$) as described in Fig. 1.
