# Supplementary material for: Coregulation of glutamine synthetase1;2 (GLN1;2) and NADH-dependent glutamate synthase (GLT1) gene expression in Arabidopsis roots in response to ammonium supply
Source: Front Plant Sci. 2023 Feb 20;14:1127006. doi: 10.3389/fpls.2023.1127006 (PMC9986259; doi:10.3389/fpls.2023.1127006)
Supplement: Supplementary file 2 [file Presentation_2.pdf]

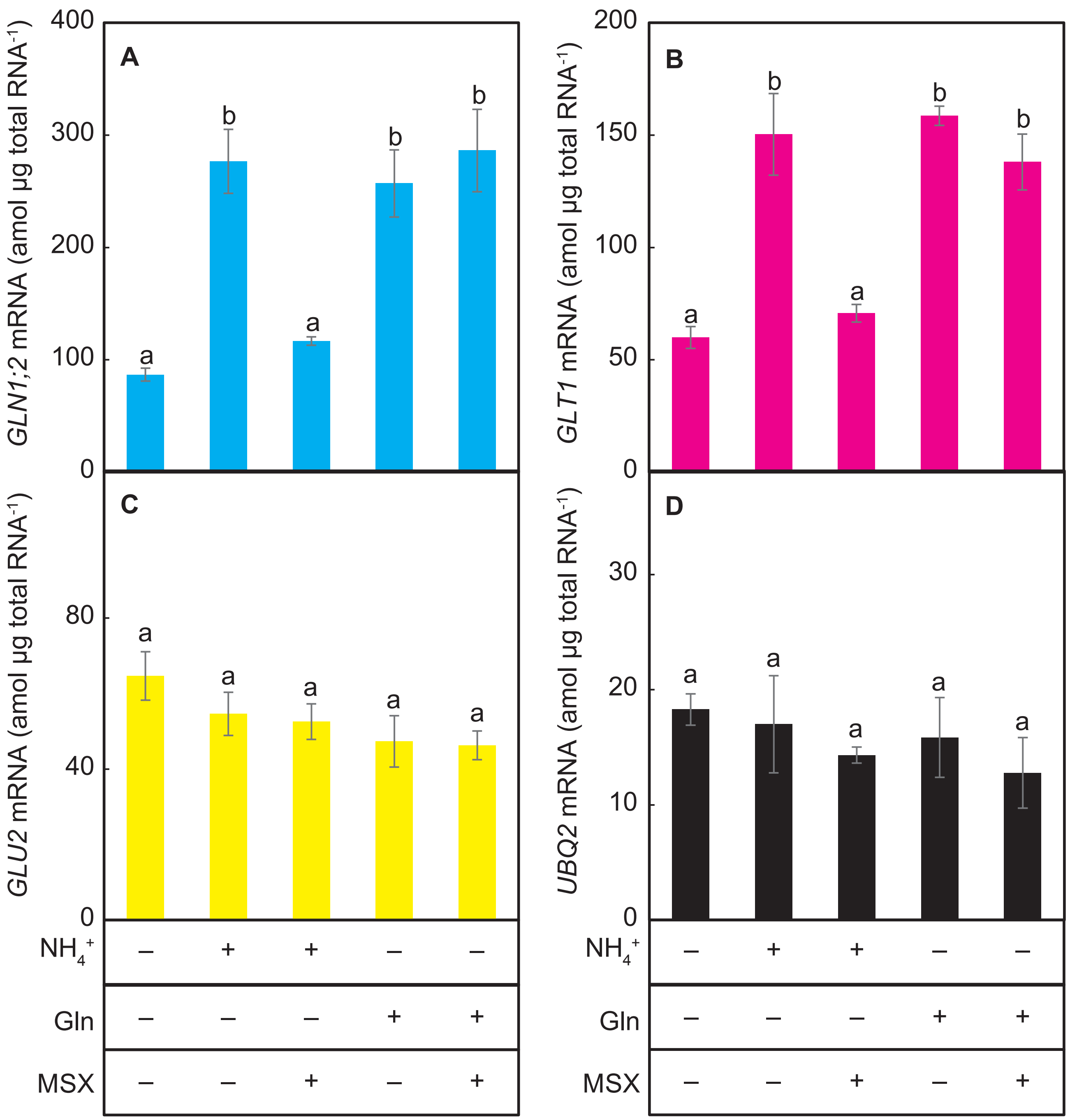


**Figure S2.** ***GLN1;2*** **and** ***GLT1*** **response to ammonium is caused by glutamine or post-glutamine metabolites**

Two-week-old Arabidopsis plants (accession Col-0) grown on MGRL agar media were subjected to nitrogen starvation for 3 days prior to the treatment. Pre-cultured plants were treated for 6 hours on modified MGRL media without nitrogen, or with 10 mM ammonium ($NH_4^+$) or with 5 mM glutamine as the sole nitrogen source, and with or without 5 mM methionine sulfoximine (MSX) as a GS inhibitor. Gene expression levels were quantified by qPCR for *GLN1;2* (a, cyan column), *NADH-GOGAT* (b, *GLT1*, magenta column), *Fd-GOGAT2* (c, *GLU2,* yellow column), and *UBQ2* as the internal control (d, black column). One-way ANOVA analysis was performed and followed by Bonferroni as post-hoc. Letters denote statistically significant differences between the treatment groups and no nitrogen control.
