# Supplementary material for: Coregulation of glutamine synthetase1;2 (GLN1;2) and NADH-dependent glutamate synthase (GLT1) gene expression in Arabidopsis roots in response to ammonium supply
Source: Front Plant Sci. 2023 Feb 20;14:1127006. doi: 10.3389/fpls.2023.1127006 (PMC9986259; doi:10.3389/fpls.2023.1127006)
Supplement: Supplementary file 3 [file Presentation_3.pdf]

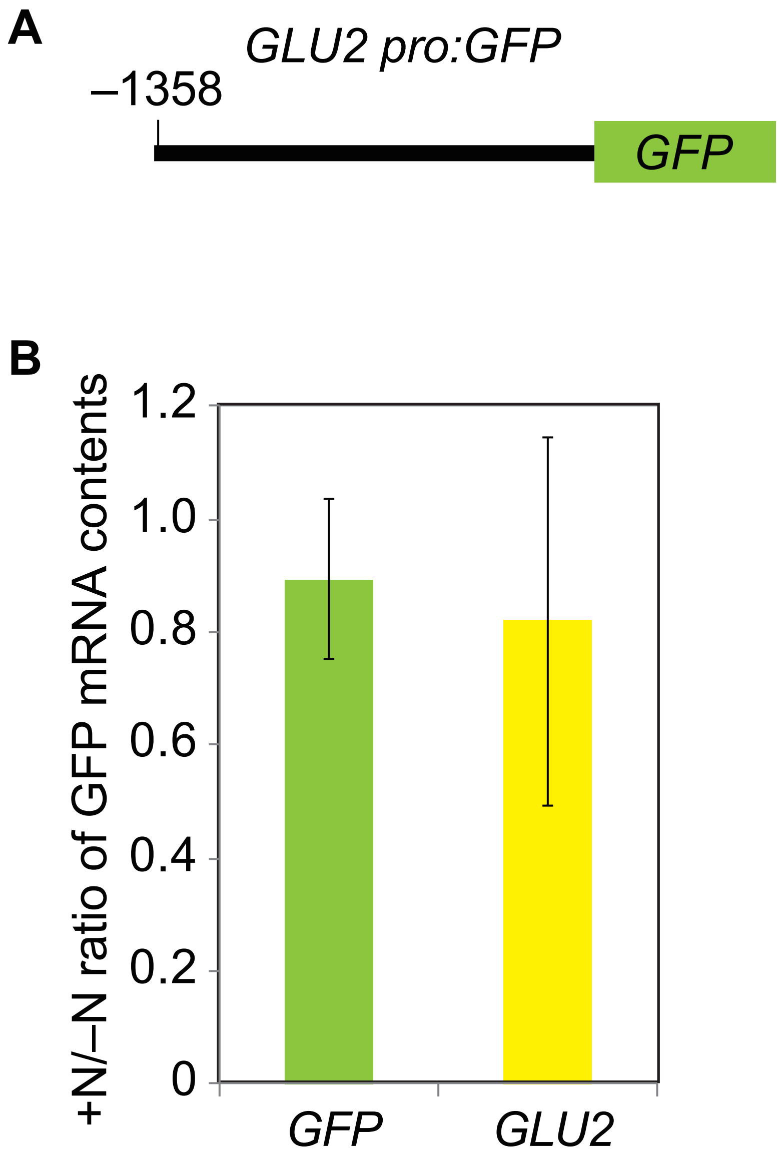

Figure S3. Ammonium response of *GLU2* promoter activity.

(a) Schematic chart of the promoter:GFP construct with a 1,358-bp promoter region upstream of the translation initiation site of *GLU2*.

(b) RT-qPCR analysis of *GLU2* and *GFP* mRNA levels in root tissues of the *GLU2* promoter:GFP line. The ammonium-response of *GLU2* (yellow column) and *GFP* (green column) transcripts were determined based on their relative abundance between the ammonium-treated and no nitrogen control samples ($+NH_4^+$/–N). Plants were treated with or without ammonium as described in Fig. 1.
